# Poultry meat freshness evaluation using electronic nose technology and ultra-fast gas chromatography

**DOI:** 10.1007/s00706-017-1969-x

**Published:** 2017-07-01

**Authors:** Wojciech Wojnowski, Tomasz Majchrzak, Tomasz Dymerski, Jacek Gębicki, Jacek Namieśnik

**Affiliations:** 1 Department of Analytical Chemistry, Faculty of Chemistry, Gdańsk University of Technology, Gdańsk, Poland; 2 Department of Chemical and Process Engineering, Faculty of Chemistry, Gdańsk University of Technology, Gdańsk, Poland

**Keywords:** Meat quality, Gas chromatography, Odoriferous substances, Sensors, Electronic olfaction

## Abstract

**Abstract:**

To ensure that chicken meat products are safe to consume, it is important to be able to reliably determine its shelf-life. To assess the applicability of ultra-fast gas chromatography and electronic nose technology in evaluation of poultry, an analysis of the headspace of ground chicken meat samples refrigerated over a period of 7 days was performed. Chemometric techniques were used to mine additional information from a multiparametric data set. As a reference, sensory evaluation was also conducted, and several volatile chemical compounds that can potentially be used as poultry meat decomposition indicators were identified. The obtained results suggest the possibility of using both techniques to supplement the established methods of chicken meat quality assessment.

**Graphical abstract:**

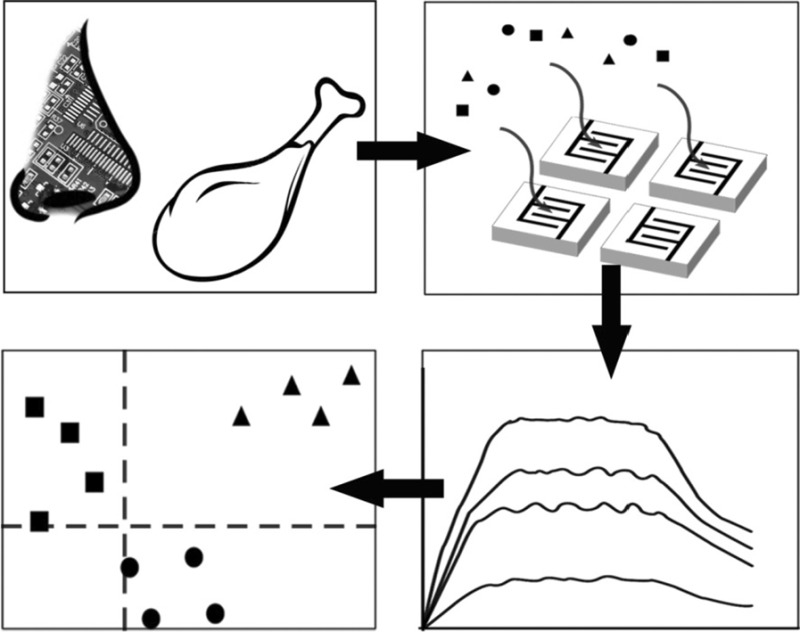

## Introduction

Poultry which constitutes 36% of the global meat consumption is an important element of many diets due to a high content of easily digestible protein, as well as vitamins and microelements necessary for maintaining proper metabolic processes. Experts at the Organisation for Economic Co-operation and Development estimate that its overall consumption shall increase by 20% in the next 10 years [1]. In the sector of chicken meat production and processing there are numerous challenges pertaining to the evaluation of product quality, both at the industrial [2] and retail level [3]. The main factors that are taken under consideration by consumers when choosing a meat product are colour and aroma. However, simple sensory evaluation of hedonistic qualities is often not enough to determine, with reasonable certainty, whether the protein in question is spoiled or not. In the industry, there are several methods of assessing the quality of poultry meat. Most commonly, samples are inspected by a trained sensory expert who evaluates the sample’s appearance and aroma. However, these specialists usually cannot work for more than 3–4 h at a time due to sensory fatigue. The gold standard in determining the shelf-life of chicken meat is the analysis of the total count of bacteria [4]. In the case of this approach an incubation period of up to 72 h is required, which means that the product leaves the plant before its quality can be established. Moreover, this type of analysis often fails to indicate the presence of psychrotrophic bacteria which proliferate in chicken meat during cold storage [5]. A quick and inexpensive technique is needed that would supplement the currently used methods by providing a reliable prediction of the product’s shelf-life and overall quality.

For that reason, a device equipped with a chemical sensor sensitive to a particular spoilage indicator (‘marker’) would prove useful. Unfortunately, no single compound has yet been identified as the one primarily responsible for the aroma of meat [6]. On the other hand, a combination of volatile compounds may form a unique “fingerprint”, an aroma profile, which may be used as an indicator of spoilage or to differentiate between types of meat. Devices called electronic noses, comprised of an array of chemical sensors coupled with data processing and pattern recognition algorithms can be employed with good results in this area [7–10].

The potential use of chemical sensor-based electronic noses for evaluation of raw chicken meat was investigated by several researchers. Galdikas et al. used an array of metal oxide sensors to investigate the freshness of poultry [11]. They considered the signal o in the transient state, treated as an exponential decrease (‘the time constant approach’), from the onset of sampling, as input data for pattern recognition, as opposed to only gathering signals after the sensors have been saturated. They concluded that the data displayed greater variance in the former case, which aids in subsequent correct recognition using data processing. A commercial electronic nose was used for quality control of modified atmosphere packaged chicken in various temperature regimes [3]. Good relationship was found with the results of microbiological analysis. In particular, PLS regression models predicting the counts of *Enterobacteriaceae* and of hydrogen sulfide-producing bacteria in the packaged chicken samples have shown correlation above 0.9. In a different approach, a colorimetric sensor array with a specific calorific fingerprint to volatile compounds was used to classify samples of chicken fillet refrigerated over a period of 9 days, using TVB-N as Ref. [12]. Discrimination rates of 87–100% depending on classification model were obtained for two classes, namely ‘fresh’ and ‘stale’. It should be noted that the electronic olfaction technique is constantly developing and its applications are much wider, than just in food quality assessment [13, 14]. Potential applications of e-noses include, among others, environmental odour monitoring [15–17], medical diagnostics [18, 19], and telemedicine [20].

Another analytical technique used for rapid acquisition and analysis of the sample’s headspace is ultra-fast gas chromatography (ultra-fast GC), which has been shown to be applicable to trace and ultra-trace analysis of volatile and semi-volatile compounds, also with narrow bore columns and difficult sample matrices such as food [21]. It was introduced to curtail the time of a single analysis whilst retaining the possibility of simultaneous separation and identification of chemical compounds. Ultra-fast gas chromatography was previously used for classification of pork based on feed composition [22], and for differentiation of fresh, refrigerated, and frozen pork neck [23, 24].

The above-mentioned techniques can be used to determine if a given sample of meat can be safely consumed. In this paper we evaluate the applicability of electronic nose technology and ultra-fast gas chromatography coupled with chemometrics in the evaluation of the freshness of refrigerated poultry meat.

## Results and discussion

**Fig. 1:**
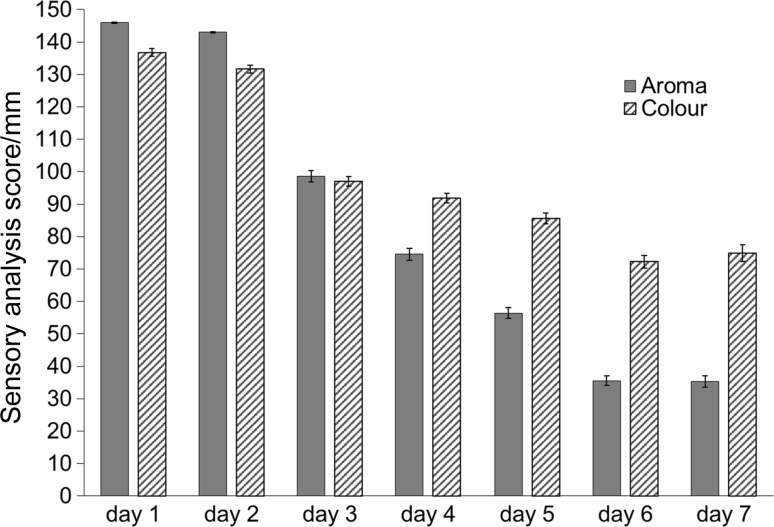
Results of sensory evaluation of refrigerated poultry meat samples

To obtain a reference for the evaluation of the applicability of instrumental techniques in chicken meat freshness assessment a sensory panel was first conducted. It is a well-established method of estimation of food product’s quality and can produce reliable results if the test is conducted in accordance with a proper procedure. Its main advantage lies in the fact that, since it can be used to directly specify hedonic qualities of samples, a good estimation of the consumer’s experience can be obtained. A panel of 12 females and males aged 20–27 was asked to evaluate both the aroma and the appearance of refrigerated meat samples on 7 consecutive days. The obtained sensory scores are plotted in Fig. 1. The first aroma traits perceived as undesirable were observed after the second day of storage, and a significant drop in the test scores took place after the fourth day. The samples refrigerated for 6 and 7 days were perceived as spoiled based on the aroma. It should be noted that the panellists did not determine that the samples were spoiled even after the seventh day based solely on the appearance.

**Fig. 2:**
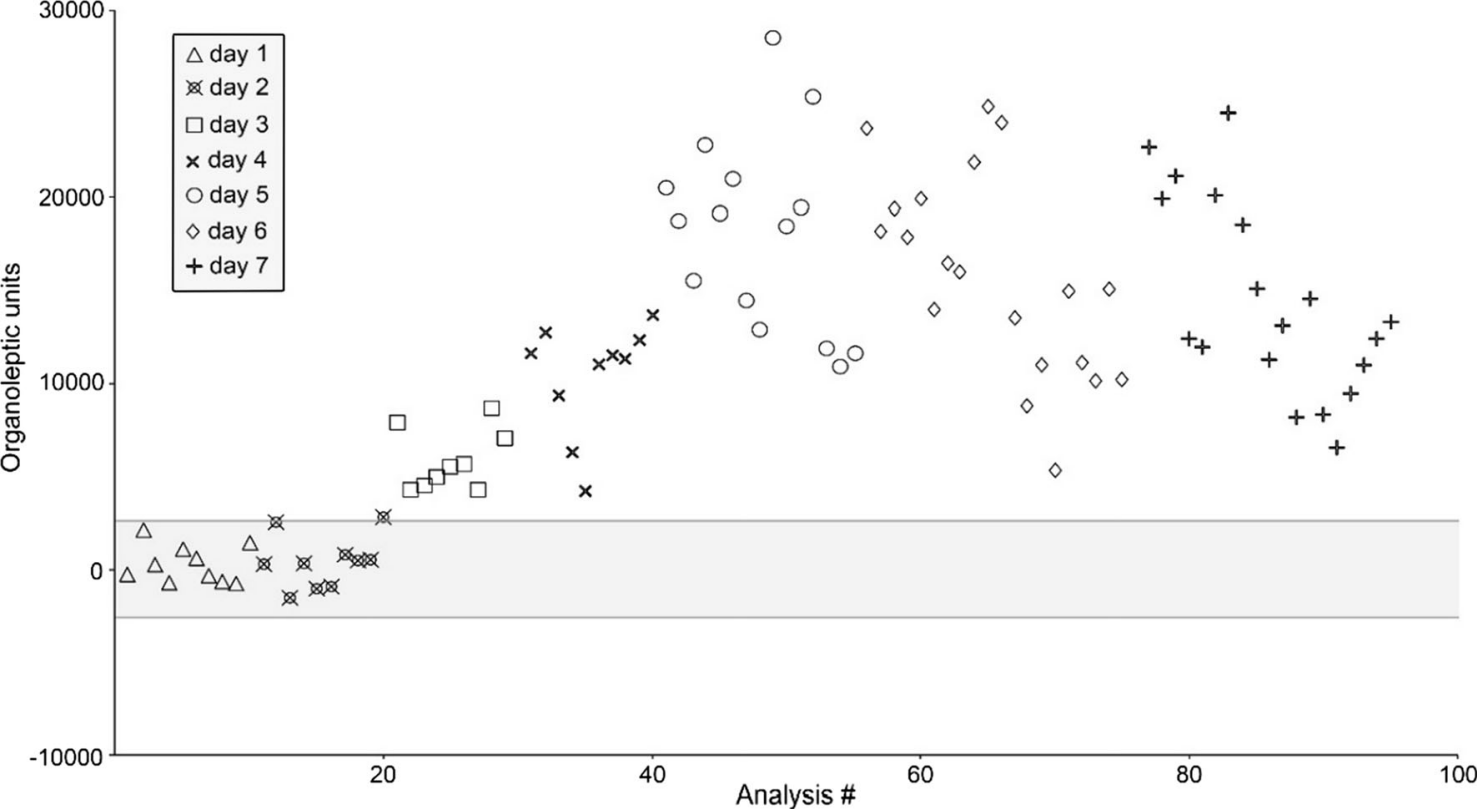
Results of SQC analysis of poultry meat samples

The changes in the aromatic profile were monitored using ultra-fast GC. Chromatograms obtained from both flame ionization detectors (FID) were processed holistically, as “fingerprints”. During the process of meat spoilage the composition of the sample’s volatile fraction changes, mostly due to bacterial activity. As this bacterial decomposition gives rise to numerous chemical compounds that were previously not present in the sample, chromatographic fingerprint changes can be used for the purpose of classification using chemometrics. For that purpose, statistical quality analysis (SQC) method was used. It is often employed in the industry, as it outputs a binary information based on the reference class—in this instance whether a given sample is statistically similar to the samples analysed on day 1. In this statistical method several conforming samples are analysed to calculate an average value and standard deviation from each data point. The complex data are then simplified into a single variable using multivariate analysis, and a difference between conforming training samples and the unknown sample is expressed as distance (in this case organoleptic unit). The control limit, or ‘envelope of confidence’ is calculated based on standard deviation of training samples [25]. Based on the results, which are depicted in Fig. 1, it can be assessed that noticeable differences in the sample’s aroma profile (results beyond the envelope of confidence) occur after the second day of storage, and the differences both between and within the classes become significant after the fourth day of storage (Fig. 2).

**Fig. 3:**
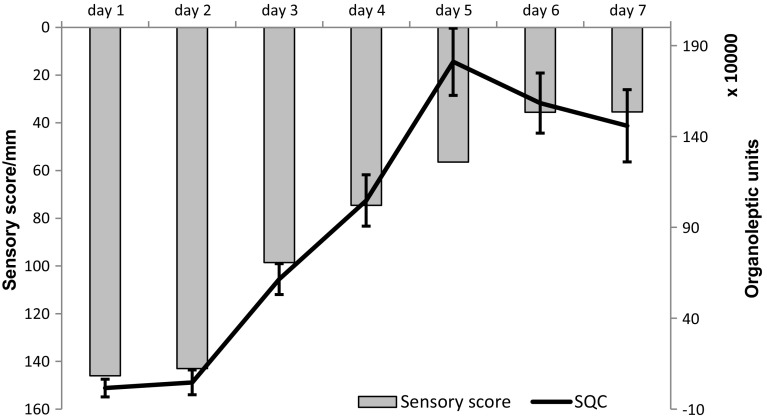
Results of sensory evaluation (inverted for clarity) and SQC analysis

These data correlate well with sensory analysis results (*R* ^2^ = 0.915 for averaged daily scores). The results of sensory evaluation and SQC analysis are juxtaposed in Fig. 3.

Ultra-fast GC can also be used for qualitative identification of volatile compounds present in the sample’s headspace. Listed in Table 1 are the compounds which displayed the greatest relative changes in concentration over the span of research, which in turn had the greatest impact on the statistical analysis. It is important to note that these compounds are not necessarily the ones at the highest concentration levels in the samples’ volatile fraction, but the ones for which the corresponding chromatographic peaks display the greatest variance over the period of seven days. These compounds can be considered potential indicators of chicken meat spoilage. Also given in Table 1 are odour thresholds of these compounds in water [26–33].

**Table 1:** Some compounds identified as potential poultry meat spoilage indicators

Chemical compound	Sensory descriptor	Kovats indices MXT-5/MXT-1701	Odour threshold/ppm
Butan-2-one	Butter, cheese, chemical	587/685	50000
Ethyl acetate	Rancid, buttery, fruity	609/673	5
Heptane	Fruity, sweet	700/700	400
3-Methyl-1-butanol	Bitter, burnt	728/842	250–300
2,4-Octadiene	Glue, warm	816/825	–
Toluene	Fruity, caramelized, paint	765/819	0.17
Pyridine	Cold meat fat, fishy, rancid	736/834	2000
Dimethyl sulfide	Cabbage, boiled vegetables	484/559	0.3–1
Ethyl butyrate	Fruity, sweet, pineapple	756/813	1
Diacetyl	Caramelized, buttery, fruity, alcoholic	571/683	2.3–6.5

**Fig. 4:**
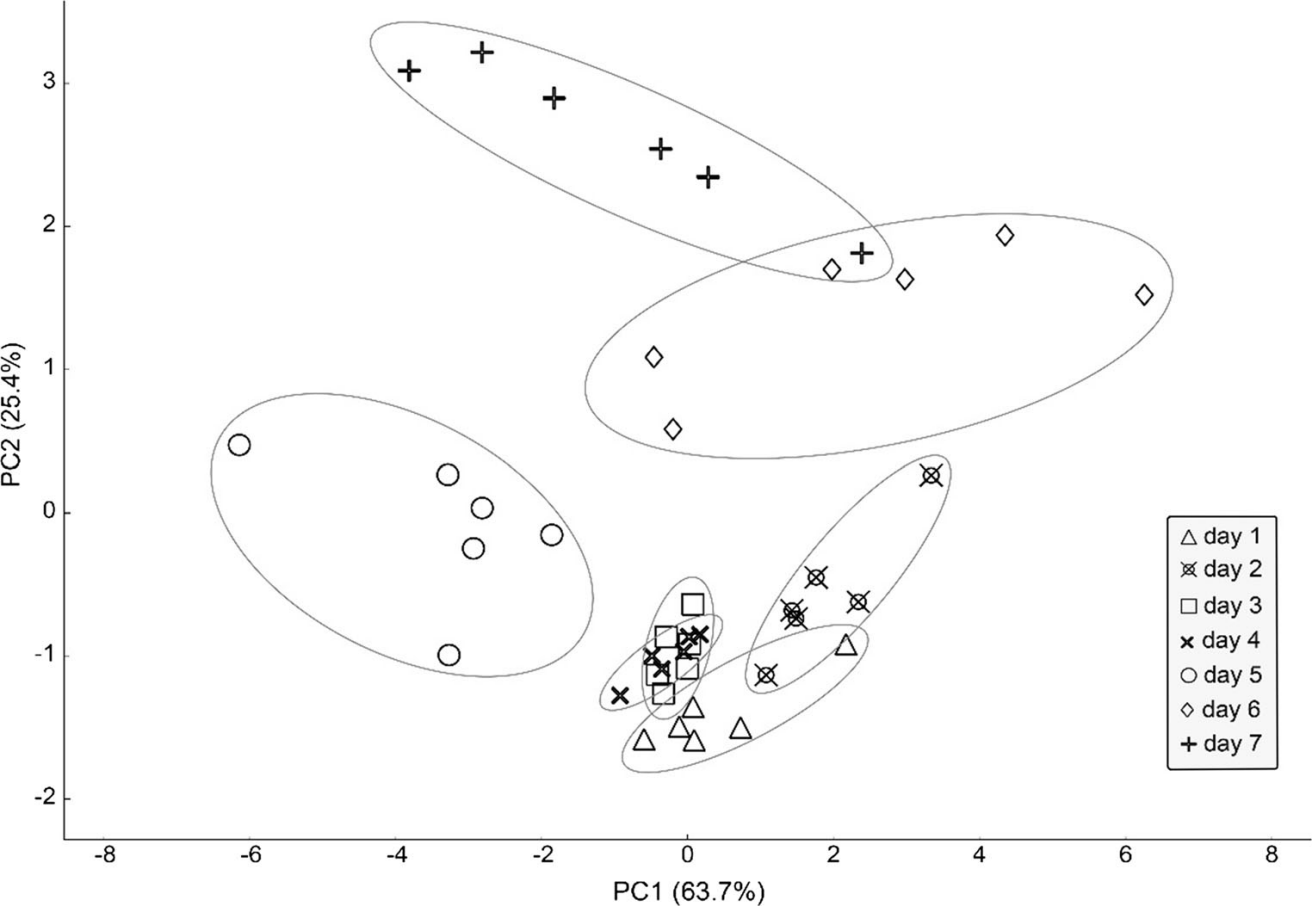
Classification of refrigerated poultry samples using PCA

Despite its obvious advantages like high sensitivity and relatively short time of a single analysis, ultra-fast GC remains relatively expensive to use. For implementation in medium- and small-size poultry processing plants, in wholesale and, in the foreseeable future at retail and consumer level an electronic nose equipped with an array of chemical sensors might in the future prove more cost-effective, especially when issues with sensor drift [34], recovery [35], susceptibility to fluctuations of relative humidity, and sensor poisoning [10] are resolved. For that reason, the performance of an e-nose prototype with 6 metal oxide semiconductor sensors (MOS) and two photo-ionisation detectors (PID) in determining the shelf-life of chicken meat was evaluated. Principal component analysis (PCA) technique was used to reduce the data dimensionality. In this unsupervised method, a new coordinate system is positioned in a way that maximizes variance along the first new coordinate, with each succeeding component orthogonal to the previous one. The result of this operation is a new coordinate system, in which most of the variance can be explained by first few principal components. Principal component analysis is usually used to model, compress, and visualize multivariate data. In the case of the dataset obtained during the analysis of refrigerated poultry samples, the first two components explained 89.1% of the total variance, as depicted in Fig. 4. It can be observed that not unlike the results of SQC analysis of data obtained using ultra-fast GC the groups of days 5–7 are more clearly separated, with more internal variation. The groups corresponding to samples from the first four days of refrigerated storage exhibit some overlap; however, they are distinct from the samples from the remaining three days of storage. This means that although this method apparently lacks the precision necessary to successfully classify the samples into particular days of refrigeration, it can be precise enough to determine, whether a consumer would deem the sample acceptable or not.

To predict the shelf-life of a meat product with better precision and in a more conclusive way supervised chemometric methods are used. Pre-processed data obtained using the electronic nose prototype were analysed with several supervised algorithms to determine the use of which one leads to best results. The prediction power of these algorithms was evaluated using tenfold cross validation, and the results are listed in Table 2. Support vector machine (SVM) method performed the best, with 0.786 classification accuracy (CA) and precision of 0.789. However, classification accuracy was over 97% when the target classes were reduced to just two: “acceptable” (day 1–4) and “unacceptable” (day 5–7) or “fresh” (day 1–2) and “suspect” (day 3–7), based on the sensory evaluation.

**Table 2:** Cross validation of supervised algorithms used for classification of data from the analysis of stored poultry

Method	CA	Precision
k-Nearest neighbour	0.667	0.646
Classification tree	0.524	0.570
SVM	0.786	0.789
Logistic regression	0.571	0.575
Naïve Bayes	0.500	0.526
Random forest	0.619	0.647

## Conclusion

The currently used methods of assessing chicken meat shelf-life have several limitations, including high cost and a long time of a single analysis. There is a need to develop a technique which would supplement these methods and the use of which would provide a quick and reliable evaluation of the meat’s quality before it leaves the processing plant. This can possibly be done by implementing ultra-fast GS, which would allow for a quick and reliable prediction of the product’s shelf-life. When cost is an issue a dedicated electronic nose equipped with an array of chemical sensors can possibly be used. Chemical sensors lack the sensitivity of detectors used in gas chromatography, they can, however, be used to obtain reliable results when employed together with proper chemometric analysis of their response signals.

Based on the presented research it can be concluded that the headspace analysis of chicken meat samples can be a valid way of assessing their freshness. Bacterial spoilage leads to noticeable changes in the composition of the volatile fraction of samples stored under refrigeration, which allows for classification of these samples using the “fingerprint” method. This can be reliably done using ultra-fast GC coupled with chemometrics, and also using the electronic nose technique, especially when only a binary estimation is needed, e.g. “fresh”/”suspect”. The results obtained in this way can then be correlated with those obtained using more established methods, like sensory evaluation or bacterial analysis. Still, in this article presented are the results of a preliminary study. To obtain a more detailed insight into the possibility of using ultra-fast GC and electronic olfaction for raw meat quality assessment. A more extensive investigation would involve samples obtained from various sources and refrigerated in different temperatures. It would be beneficial to analyse the sample’s headspace with conventional gas chromatography–mass spectrometry to obtain a more definite information regarding its composition, and also to correlate the results with bacterial counts—a method more often used in the industry than sensory panels.

In the author’s opinion commercial e-nose devices dedicated to meat quality assessment will be introduced in the near future, possibly even in a hand-held format suitable for the consumer market despite the fact that meat is a relatively complex matrix and some issues regarding the sensor’s long-term reliability and sensitivity are yet to be resolved.

## Experimental

### Sample preparation

Poultry samples were obtained from a local distribution centre in Gdańsk, Poland. The chickens were slaughtered in the evening on the day preceding the first day of the analysis and transported under refrigeration at 2.4 °C to the distribution centre, where the carcasses were dismembered and the breast muscle minced in the morning of the first day of analysis. Samples weighing 3 g were placed in 20 cm^3^ headspace vials, covered with plastic wrap, and refrigerated at 4 °C. Prior to analysis the vials were sealed with caps lined with a silicon-PTFE membrane and incubated for 5 min at 40 °C. The samples were analysed over the period of seven consecutive days, with a new sample used for every analysis.

### Sensory panel

The panel consisted of 12 members, 8 female and 4 male, aged 20–27. The panellists were asked to assess first the appearance, and then the odour of a sample they were presented with. They then graded the perceived hedonic quality on a 150-mm-long axis, with 150 and 0 denoting the most and least desirable qualities, respectively. The scores were then measured using callipers and averaged. After scoring panel members were also asked to comment on whether or not they would deem the sample acceptable for consumption.

### Ultra-fast gas chromatography

Headspace analysis of meat samples was performed using an ultra-fast gas chromatography unit Heracles II equipped with the HS100 autosampler (Alpha M.O.S., Toulouse, France). The device was equipped with two parallel 10-m columns packed with MXT-5 and MXT-1701 stationary phases, respectively, and with two flame ionization detectors (μFID). Samples were incubated at 40 °C for the duration of a single analysis (90 s) Hydrogen was used as carrier gas. AlphaSoft 12.4 software was used to process the data.

### Electronic nose prototype

The prototype e-nose was equipped with 6 different metal oxide sensors: TGS813, TGS816, TGS832, TGS2600, TGS2611, and TGS2620 (Figaro, Arlington Heights, USA) as well as with two photo-ionisation detectors (Ion Science, Fowlmere, UK). Samples were incubated at 40 °C for the duration of a single analysis cycle, which took 9 min (3 min purging/3 min exposure to sample/3 min purging). Carrier gas was supplied from a zero gas system (NGA 19S, Umwelttechnik MCZ GmbH, Bad Nauheim, Germany) and passed through a humidity stabilisation chamber to obtain a reliable base line. Data processing was performed using Orange v.2.7 software (Bioinformatics Lab, University of Ljubljana, Slovenia) [36].
